# Complex Interplay between the Lipin 1 and the Hepatocyte Nuclear Factor 4 α (HNF4α) Pathways to Regulate Liver Lipid Metabolism

**DOI:** 10.1371/journal.pone.0051320

**Published:** 2012-12-07

**Authors:** Zhouji Chen, Matthew C. Gropler, Mayurranjan S. Mitra, Brian N. Finck

**Affiliations:** Department of Medicine, Washington University School of Medicine, St. Louis, Missouri, United States of America; Nihon University School of Medicine, Japan

## Abstract

Lipin 1 is a bifunctional protein that serves as a metabolic enzyme in the triglyceride synthesis pathway and regulates gene expression through direct protein-protein interactions with DNA-bound transcription factors in liver. Herein, we demonstrate that lipin 1 is a target gene of the hepatocyte nuclear factor 4α (HNF4α), which induces lipin 1 gene expression in cooperation with peroxisome proliferator-activated receptor γ coactivator-1α (PGC-1α) through a nuclear receptor response element in the first intron of the lipin 1 gene. The results of a series of gain-of-function and loss-of-function studies demonstrate that lipin 1 coactivates HNF4α to activate the expression of a variety of genes encoding enzymes involved in fatty acid catabolism. In contrast, lipin 1 reduces the ability of HNF4α to induce the expression of genes encoding apoproteins A4 and C3. Although the ability of lipin to diminish HNF4α activity on these promoters required a direct physical interaction between the two proteins, lipin 1 did not occupy the promoters of the repressed genes and enhances the intrinsic activity of HNF4α in a promoter-independent context. Thus, the induction of lipin 1 by HNF4α may serve as a mechanism to affect promoter selection to direct HNF4α to promoters of genes encoding fatty acid oxidation enzymes.

## Introduction

The control of hepatic intermediary metabolism is critical to maintaining systemic energy homeostasis. For example, during conditions of nutrient scarcity (fasting), the liver takes up and oxidizes fatty acids to provide the brain and other peripheral tissues with ketone bodies and uses the chemical energy stored in fat to drive gluconeogenesis. The liver also provides lipid to other peripheral tissues by esterifying fatty acids into triglycerides (TG) and secreting them in the form of very low density lipoproteins (VLDL). Complex regulatory mechanisms have evolved to control hepatic fatty acid utilization, trafficking, and export. However, nutrient excess and obesity perturb the ability of the liver to maintain homeostasis and these hepatic metabolic abnormalities contribute to the hyperglycemia and dyslipidemia that are prevalent in type 2 diabetes mellitus.

Recent work has demonstrated that the lipin family of proteins (lipin 1, 2, and 3) are critical regulators of hepatic intermediary metabolism [1] that are strongly affected by alterations in energy homeostasis [2], [3]. Lipins are bifunctional intracellular proteins that regulate fatty acid metabolism at two distinct regulatory levels. Lipins act as phosphatidic acid phosphohydrolase (PAP) enzymes that catalyze the dephosphorylation of phosphatidic acid (PA) to generate diacylglycerol (DAG); the penultimate step in triglyceride (TG) synthesis [4], [5], [6]. Unlike other enzymes in the TG synthetic pathway that are integral membrane proteins, lipins are soluble and contain a nuclear localization signal [7], [8], [9]. Lipins also act as transcriptional regulatory proteins by associating with DNA-bound transcription factors to modulate their activity [7], [10], [11]. In liver, lipin 1 interacts with and coactivates the peroxisome proliferator-activated receptor α (PPARα) and its coactivator (PPARγ coactivator 1α (PGC-1α)) to enhance the expression of genes involved in fatty acid oxidation by recruiting in other coactivator proteins with histone acetyltransferase activity [10]. The effects of lipin 1 on hepatic fatty acid oxidation can proceed independent of PPARα, but not PGC-1α [10], suggesting that other transcription factor partners of PGC-1α are also involved in this response.

Hepatic lipin 1 expression is robustly induced in liver by food deprivation in a PGC-1α-dependent manner [10]. The induction of lipin 1 by fasting likely serves to enhance fatty acid catabolism under fasting conditions since knockdown of lipin 1 by shRNA markedly attenuates the fasting-induced increase in the expression of fatty acid oxidation enzymes. Conversely, forced lipin 1 overexpression increases the expression of these enzymes and stimulates hepatic ketone production [10]. Mice with a genetic defect in lipin 1 (fatty liver dystrophic (*fld*) mice) exhibit a severe hepatic steatosis characterized by marked reductions in the expression of fatty acid oxidation enzymes [10]. Thus, lipin 1 appears to be a critical regulator of hepatic fatty acid utilization.

While it is clear that lipin 1 is a direct target gene of PGC-1α, the other components of the transcriptional complex that cooperate with PGC-1α to regulate lipin 1 expression remain unclear. Herein, we demonstrate that PGC-1α works with the hepatocyte nuclear factor 4α (HNF4α) to regulate of lipin 1 expression in liver cells. We also show that the induction of lipin 1 feeds forward to modulate HNF4α activity in a promoter-specific manner to direct this nuclear receptor to activate hepatic fatty acid oxidation while suppressing expression of genes encoding apoproteins. These data further elucidate the regulatory mechanisms by which lipin 1 controls hepatic metabolism and suggest that the transcriptional regulatory function of this protein serves to fine-tune hepatic metabolic control.

## Methods

### Mouse Studies

All animal experiments were approved by the Animal Studies Committee of Washington University School of Medicine. C57BL/6 mice were generated from a colony established in the Washington University mouse facility. Mice constitutively deficient in lipin 1 (*fld* mice), were compared to wild-type (+/+) littermate control mice (Balb/cByJ strain).

### Gene Expression Analyses

For quantitative PCR studies, first-strand cDNA was generated by reverse transcription using total RNA. Real-time RT-PCR was performed using the ABI PRISM 7500 sequence detection system (Applied Biosystems, Foster City, CA) and the SYBR green kit. Arbitrary units of target mRNA were corrected by measuring the levels of 36B4 RNA.

### Mammalian Cell Culture and Transient Transfection

Primary cultures of mouse hepatocytes were prepared as described [12]. After a 2 h attachment period, hepatocytes were infected with adenovirus to drive overexpression of proteins defined below, then studied after 48 h of infection. Palmitate oxidation rates were determined using ^3^H-palmitate as previously described [2]. VLDL-TG secretion was measured using ^3^H-glycerol after oleate stimulation (0.3 mM) as previously described [12].

### Transient Transfection and Luciferase Assays

HepG2 and HEK-293 cells were maintained in DMEM-10% fetal calf serum. Transient transfections with luciferase reporter constructs were performed by calcium-phosphate co-precipitation. SV40-driven renilla luciferase expression construct was also included in each well. For all vectors, promoterless reporters or empty vector controls were included so that equal amounts of DNA were transfected into each well. Luciferase activity was quantified 48 h after transfection by using a luminometer and the Stop & Glo® dual luciferase kit (Promega). Assays were performed in duplicate. To control for transfection efficiency, firefly luciferase activity was corrected to renilla luciferase activity.

### Co-immunoprecipitation and Western Blotting Analyses

In co-immunoprecipitation (co-IP) experiments, HepG2 cells were lysed and incubations performed in NP40-containing lysis buffer (20 mM Tris HCl, 100 mM NaCl, 0.5% NP40, 0.5 mM EDTA, 0.5 mM PMSF, and protease inhibitor cocktail). Proteins were immunoprecipitated using protein A-conjugated agarose beads an antibody directed against HNF4α (Santa Cruz Biotechnology). Precipitated proteins were electrophoresed on acrylamide gels. Western blotting analyses for IP studies and to demonstrate overexpression of HA-tagged lipin 1 proteins were performed with mouse monoclonal anti-HA antibody (Covance). Mouse anti actin antibody was purchased from Sigma Chemical Co.

### Chromatin Immunoprecipitation (ChIP) Assays

In experiments where ChIP was the endpoint, HepG2 cells were cultured in 10 cm dishes and infected with Ad-GFP, Ad-HNF4α, and/or Ad-lipin 1β. Approximately 48 h after infection, proteins were cross-linked to chromatin by adding formaldehyde to a final concentration of 1% and incubating for 15 minutes at room temperature. Chromatin purification and ChIP assays were performed by using a commercially available ChIP assay kit (Upstate Biotechnology) according to the manufacturer’s instructions. PCR primers were designed to flank HNF4α response elements and the sequence of these primers is available upon request. For quantification, SYBR GREEN PCR was performed using DNA obtained from ChIP.

### DNA Constructs

Luciferase reporter driven by 2045 bp of the 5′ flanking region of the Lpin1 gene (−2045.*Lpin1*.Luc) was the gift of Karen Reue and the +2293.*Lpin1*.Luc, which contains 393 nucleotides of the 5′ flanking sequence, the first *Lpin1* exon (51 nucleotides), and approximately 2,242 bp of the first intron of the mouse *Lpin1* gene into a promoterless luciferase reporter vector (+2293-*Lpin1*.luc), has been recently described [13]. *Ppara*.Luc and *Acadm*.TKLuc were the gift of Bart Staels and Daniel Kelly, respectively, and have been reported and characterized previously [14], [15]. The homologous promoter-firefly luciferase reporter construct driven by the entire intergenic region between the *Apoc3* and *Apoa4* genes (*Apoc3/Apoa4.Luc*) has been described [16], [17] and was the generous gift of V. Giguerre. *Apoc3/Apoa4* promoter luciferase reporter constructs containing mutations in HNF4α response elements were derived from the parent *Apoc3/Apoa4.Luc* construct and were provided by B. Spiegelman [16]. The *Apoc3 enhancer.3X.Luc* heterologous reporter construct was driven by the thymidine kinase minimal promoter and three copies of the human *Apoc3 enhancer* (5′-GATCTCCCAGGGCGCTGGGCAAAGGTCACCTGCTGACCAGTGGAGATGAG-3′; nuclear receptor response element is underlined). The UAS-TKLuc has also been previously described [10]. The SV40-driven renilla luciferase DNA construct was obtained from Promega.

The 924 amino acid form of mouse lipin 1 (lipin 1β) fused to an N-terminal triple HA tag was overexpressed using a pCDNA3.1 vector [10]. Expression constructs driving expression of lipin 1β protein with a site-directed mutation changing isoleucine 726 and leucine 727 to phenylalanine (LXXFF) were derived from the lipin 1β construct [4], [10]. The HNF4α (pMT-HNF4α), PGC-1α, PGC-1α-mL2 (LXXLL to LXXFF), and PGC-1β expression constructs have also been described [18], [19]. The Gal4-HNF4α expression construct has also been described [20].

Adenoviral constructs to drive expression of HA-tagged lipin-1β and/or GFP [4], [10], Ad-HNF4α [16], and mutant lipin 1β (LXXFF) [2] have been previously described. Adenoviral transductions were carried out by incubating hepatocytes with adenoviruses in a 5% FBS/DMEM media overnight in 24-well plates (0.5 ml/well) or 6-well plates (2 ml/well) at an M.O.I. of 8. Experiments were carried out 40–48 h after adenoviral transduction.

### siRNA Studies

A human HNF4α-specific siRNA (siHNF4α) was obtained from Sigma. Scramble control siRNA was synthesized using a Silencer® Select siRNA kit (Ambion) as described [21]. The control siRNA and siHNF4α were transfected onto HepG2 cells using a Lipofectamine-2000 reagent (Invitrogen). At 14 hr after siRNA transfection, the cells were infected with Ad-GFP or Ad-PGC-1α and cultured for additional 34 hr and thereafter they were harvested for RNA isolation or subjected to assays measuring rates of palmitate oxidation as described above.

### Statistical Analyses

Statistical comparisons were made using analysis of variance (ANOVA) coupled to Scheffe’s test. All data are presented as means ± SEM, with a statistically significant difference defined as a *P* value <0.05.

## Results

### PGC-1α Induces Lipin 1 Expression through HNF4α in HepG2 Cells

**Figure 1:**
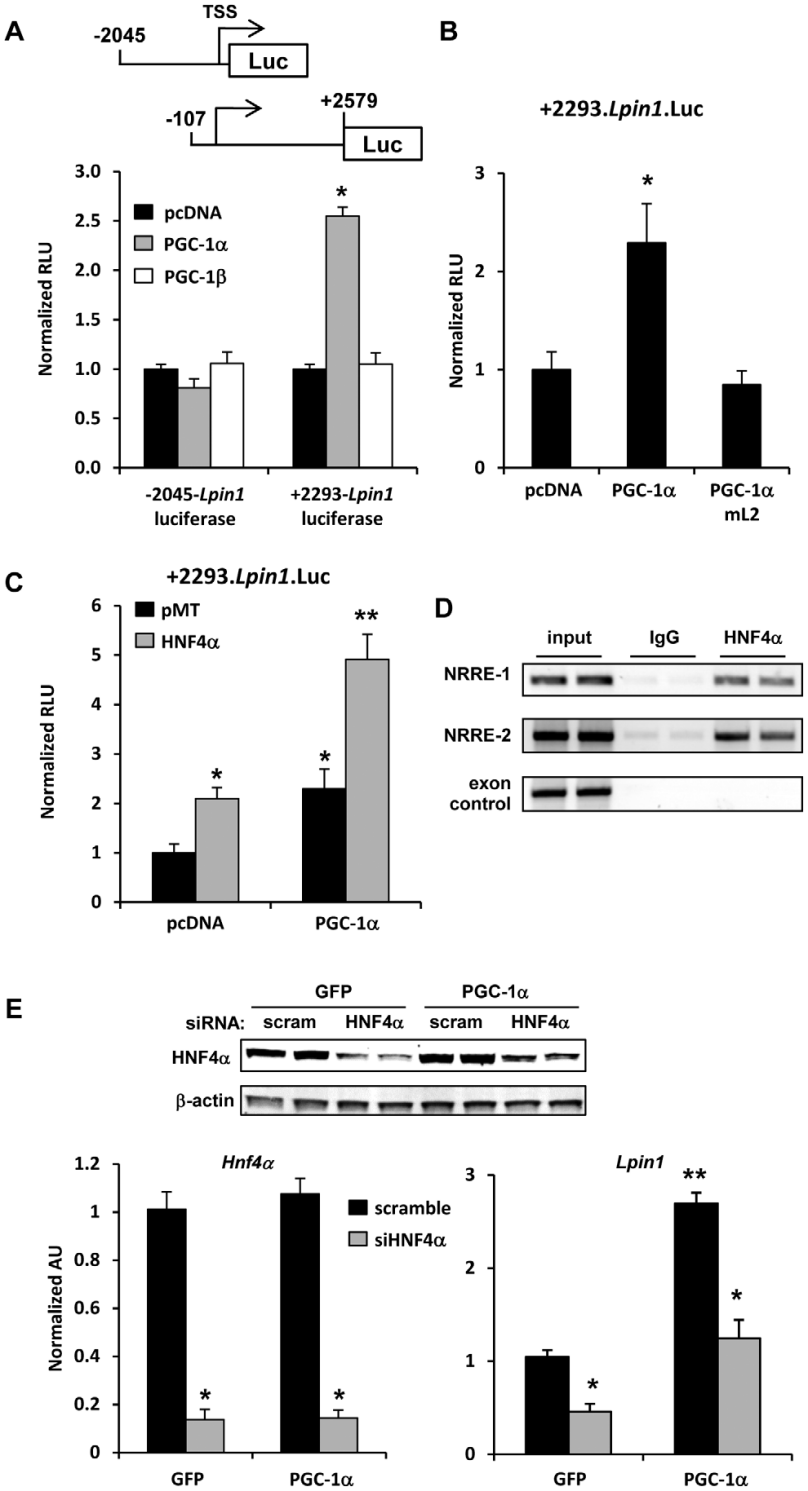
Lipin 1 is a target of HNF4α in HepG2 cells. [**A**] The schematic depicts luciferase reporter constructs driven by 2045 bp of 5′ flanking sequence or 2293 bp 3′ from the transcriptional start site of the *Lpin1* gene. Graphs depict results of luciferase assays using lysates from HepG2 cells transfected with *Lpin1.*Luc reporter constructs and cotransfected with PGC-1α or PGC-1β expression constructs as indicated. The vector values are normalized ( = 1.0). The results are the mean of 3 independent experiments done in triplicate. *p<0.05 versus pCDNA control. [**B and C**] Graphs depict results of luciferase assays using lysates from HepG2 cells transfected with +2293.*Lpin1.Luc* reporter construct and cotransfected expression constructs expressing WT or mL2 PGC-1α. The results are the mean of 3 independent experiments done in triplicate. *p<0.05 versus pCDNA control. **p<0.05 versus pCDNA control and HNF4α or PGC-1α overexpression alone. [**D**] The images depict the results of chromatin immunoprecipitation studies using chromatin from mouse hepatocytes infected with adenovirus to overexpress HNF4α. Crosslinked proteins were IP’ed with HNF4α antibody or IgG controls. “Input” represents 0.2% of the total chromatin used in the IP reactions. PCR primers were designed to amplify two regions of the *Lpin1* gene promoter containing NRREs or exon 7 (negative control). [**E**] Inset images depict results of western blotting analyses for the HNF4α and β-actin in HepG2 cells infected with adenovirus to overexpress PGC-1α or GFP (control) and transfected with siRNA to knockdown HNF4α or scramble control siRNA. Graphs depict the expression of HNF4α or lipin 1 mRNA in HepG2 cells infected with adenovirus to overexpress PGC-1α or GFP (control) and transfected with siRNA to knockdown HNF4α or scramble control siRNA (n = 6). *p<0.05 versus scramble control infected with the same adenovirus. **p<0.05 versus all other groups.

We have previously demonstrated that PGC-1α is an important regulator of lipin 1 gene expression in liver [10], but the transcription factor partners of PGC-1α that mediate this effect remain unclear. To further dissect the transcriptional mechanisms at play, we transfected HepG2 cells with expression constructs for PGC-1α or PGC-1β and *Lpin1* promoter-luciferase reporter constructs. The PGC-1α responsive region was not contained in the 2 kb 5′ flanking sequence (−2045.*Lpin1*.Luc) that is sufficient to confer responsiveness to several other transcription factors [22], [23], [24], [25]. However, PGC-1α overexpression activated a promoter driven by 393 nucleotides of the 5′ flanking sequence, the first *Lpin1* exon (53 nucleotides), and 2,240 bp of the first intron of the mouse lipin 1 gene (+2293-*Lpin1*.luc). This suggests that the PGC-1α-responsive element in HepG2 cells is contained in the first intron of the *Lpin1* gene (Figure 1A). Interestingly, PGC-1α overexpression did not affect *Lpin1* promoter activity. To begin to narrow the transcription factor partners of lipin 1 that might be mediating this response, we overexpressed PGC-1α with a site directed mutations in a leucine-rich (LXXLL to LXXFF) motif that mediates interactions with nuclear receptor partners. Mutation of the L2 domain of PGC-1α [26] was sufficient to completely block the activation of *Lpin1* promoter activity (Figure 1B). These data indicate that the ability of PGC-1α to induce lipin 1 expression depended upon a transcription factor partner, likely a nuclear receptor, which interacted with the L2 domain.

Based on these results, we hypothesized that HNF4α might be involved in this response since HNF4α requires the L2 motif of PGC-1α to interact with that coactivator [27] and interacts only weakly with PGC-1β [28]. Transfection of an expression construct for HNF4α led to a 2-fold increase in *Lpin1* promoter activity and this was additively enhanced by cotransfection of PGC-1α (Figure 1C). Chromatin immunoprecipitation studies confirmed that HNF4α was interacting with chromatin at two previously-identified [13] nuclear receptor response elements in the first intron of the *Lpin1* gene (Figure 1D), which is consistent with the promoter mapping studies. Finally, knockdown of HNF4α protein content by using siRNA led to diminished expression of *Lpin1* in HepG2 cells and abolished the induction in *Lpin1* expression caused by PGC-1α overexpression (Figure 1E).

### Lipin 1 Enhances the Effects of HNF4α on Fatty Acid Oxidation

**Figure 2:**
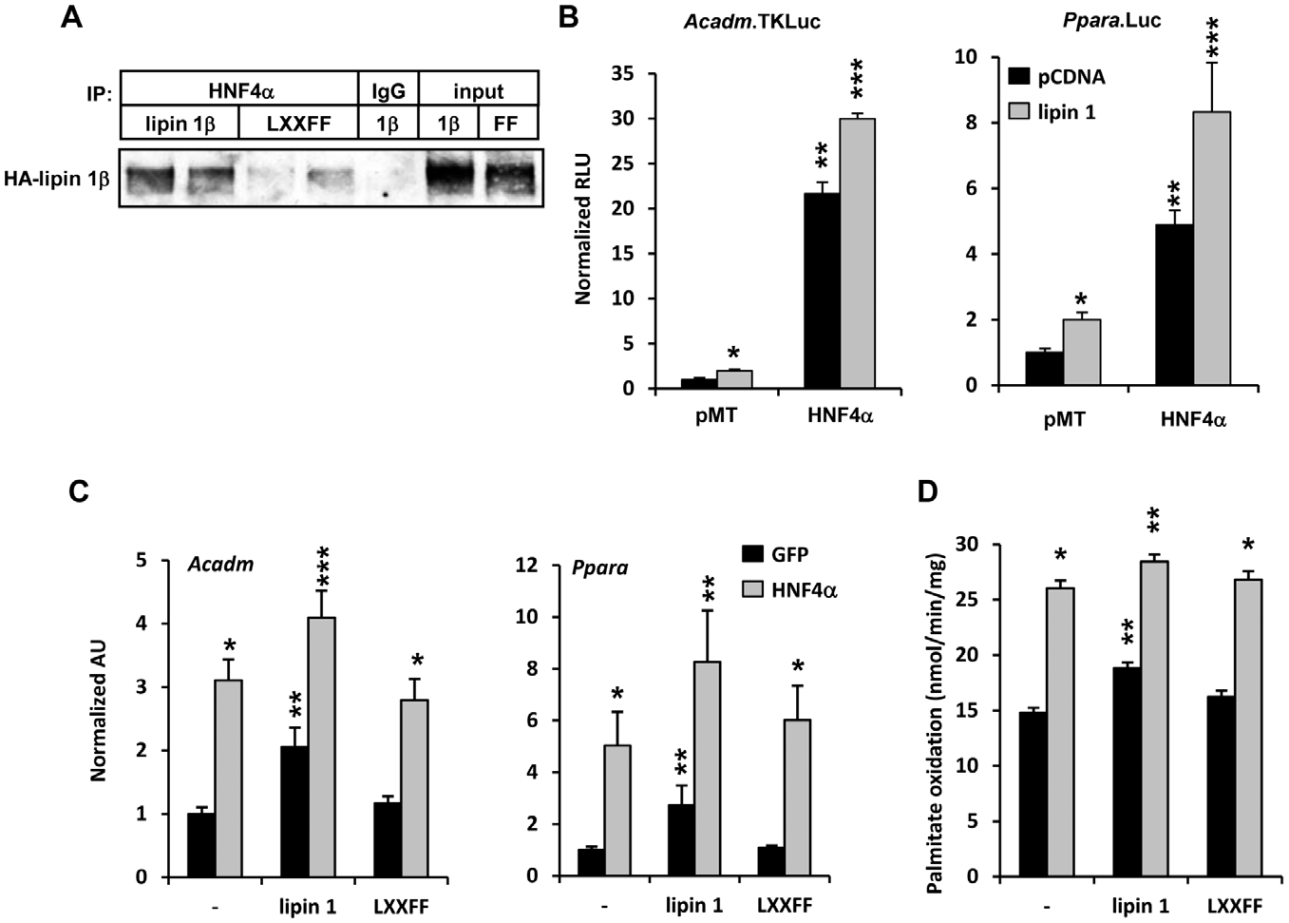
Lipin 1 enhances HNF4α-mediated increases in fatty acid oxidation. [**A**] The images depict the results of co-immunoprecipitation studies using lysates from HepG2 cells infected with adenovirus driving expression of lipin 1β or lipin 1(LXXFF). HNF4α-containing complexes were immunoprecipitated with an antibody directed against HNF4α or IgG control. Immunoprecipitated proteins were then subjected to immunoblotting with antibody directed against the HA tag of overexpressed lipin 1. Input represents 5% of the total protein used in immunoprecipitation reactions. [**B**] Graphs depict results of luciferase assays using lysates from HepG2 cells transfected with *Acadm*.TKLuc or *Ppara.*Luc and cotransfected with lipin 1 and/or HNF4α expression constructs as indicated. The results are the mean of 3 independent experiments done in triplicate. *p<0.05 versus pCDNA control. **p<0.05 versus pcDNA or lipin 1 alone. ***p<0.05 versus all other groups. [**C and D**] Primary hepatocytes were isolated from 6 week old C57BL/6 mice and infected with adenovirus driving expression of GFP or HNF4α in the presence or absence of overexpressed lipin 1β (wild-type or LXXFF). The graphs depict **[C]** the expression of *Ppara* and *Acadm* (n = 5) or [**D**] mean rates of palmitate oxidation (mean of 3 independent experiments done in triplicate) or *p<0.05 versus GFP control. **p<0.05 versus HNF4α overexpression alone. ***p<0.05 versus all other groups.

We have previously demonstrated a direct protein-protein interaction between HNF4α and lipin 1 in GST pull-down assays [10]. To confirm the interaction between HNF4α and lipin 1, HNF4α was immunoprecipitated from isolated hepatocytes overexpressing lipin 1 protein that was either wild-type or harboring a mutation in the LXXIL domain of lipin 1 that mediates its interaction with PPARα [10]. Lipin 1 was found to be immuno-coprecipitated with HNF4α and the interaction required the LXXIL motif (Figure 2A). We next evaluated the functional implications of this interaction. Lipin 1 significantly enhanced HNF4α-mediated activation of the human PPARα gene promoter-luciferase reporter and multimerized HNF4α-responsive *Acadm*-TKLuc reporter construct (Figure 2B), suggesting that lipin 1 was acting in a feed forward manner to enhance HNF4α activity. Lipin 1 overexpression augmented the effects of HNF4α on the expression of *Ppara* and *Acadm* genes (Figure 2C) and rates of fat catabolism (Figure 2D) in hepatocytes in an LXXIL-dependent manner.

**Figure 3:**
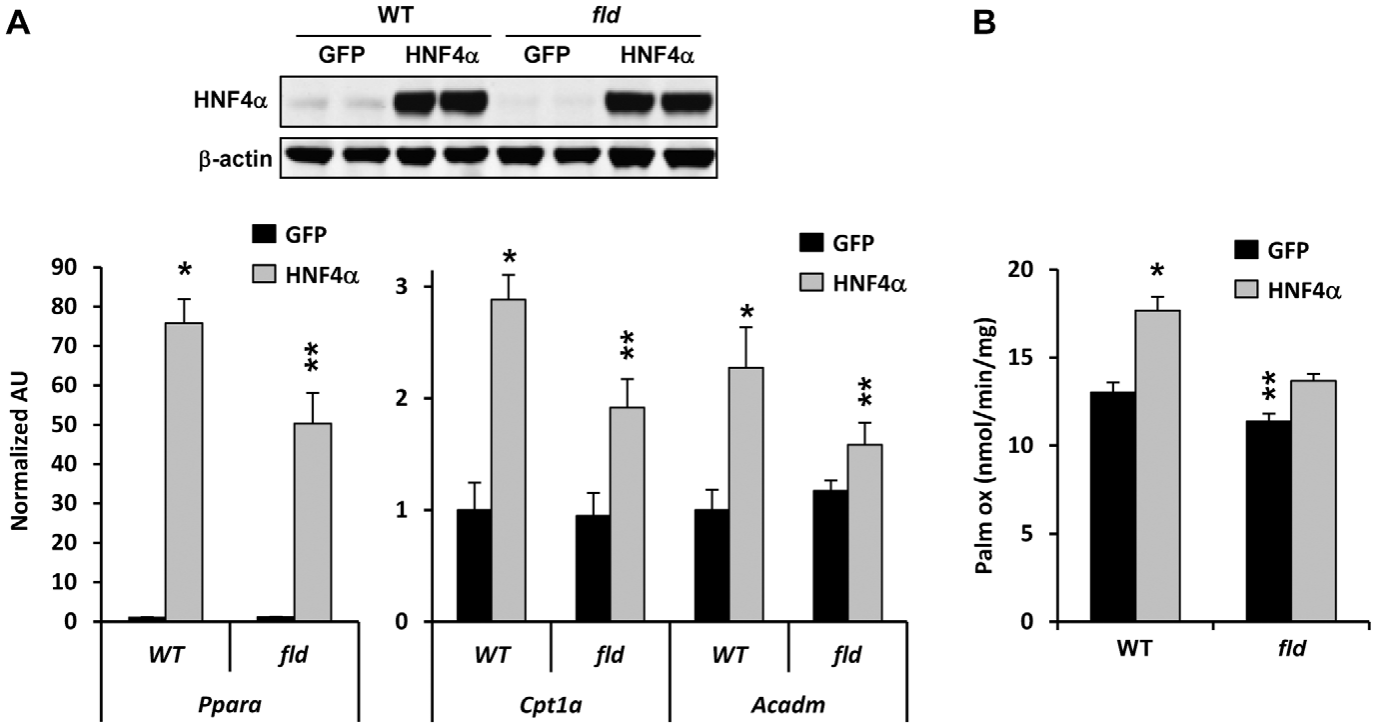
Lipin 1 deficiency impairs the ability of HNF4α to induce fatty acid oxidation. [**A**] Western blots inset above show the expression of HNF4α in hepatocytes from WT and *fld* mice infected with adenovirus to overexpress HNF4α (or GFP control). Graphs depict the expression of the indicated genes in hepatocytes from WT or *fld* hepatocytes infected with adenovirus to overexpress HNF4α or GFP (control) (n = 6). *p<0.05 versus GFP. **p<0.05 versus GFP control and WT cells expressing HNF4α. [**B**] The graphs depicts rates of palmitate oxidation in the experiment described in [A]. *p<0.05 versus WT GFP. **p<0.05 versus WT GFP and WT HNF4α.

We also took a lipin 1 loss of function approach to evaluate the interaction between lipin 1 and HNF4α. Overexpression of similar amounts of HNF4α in hepatocytes from *fld* mice, which lack lipin 1, was less effective at inducing the expression of genes encoding PPARα and fatty acid oxidation enzymes (*Cpt1a* and *Acadm*) (Figure 3A). The increase in rates of fatty acid oxidation induced by HNF4α overexpression was blunted in *fld* hepatocytes compared to WT controls (Figure 3B). Basal rates of palmitate oxidation were also diminished in *fld* hepatocytes compared to WT controls (Figure 3B). Collectively, these data indicate that lipin 1 enhances the stimulatory effects of HNF4α on fatty acid oxidation.

### Lipin 1 Suppresses the Expression of Apoproteins that are Induced by HNF4α

**Figure 4:**
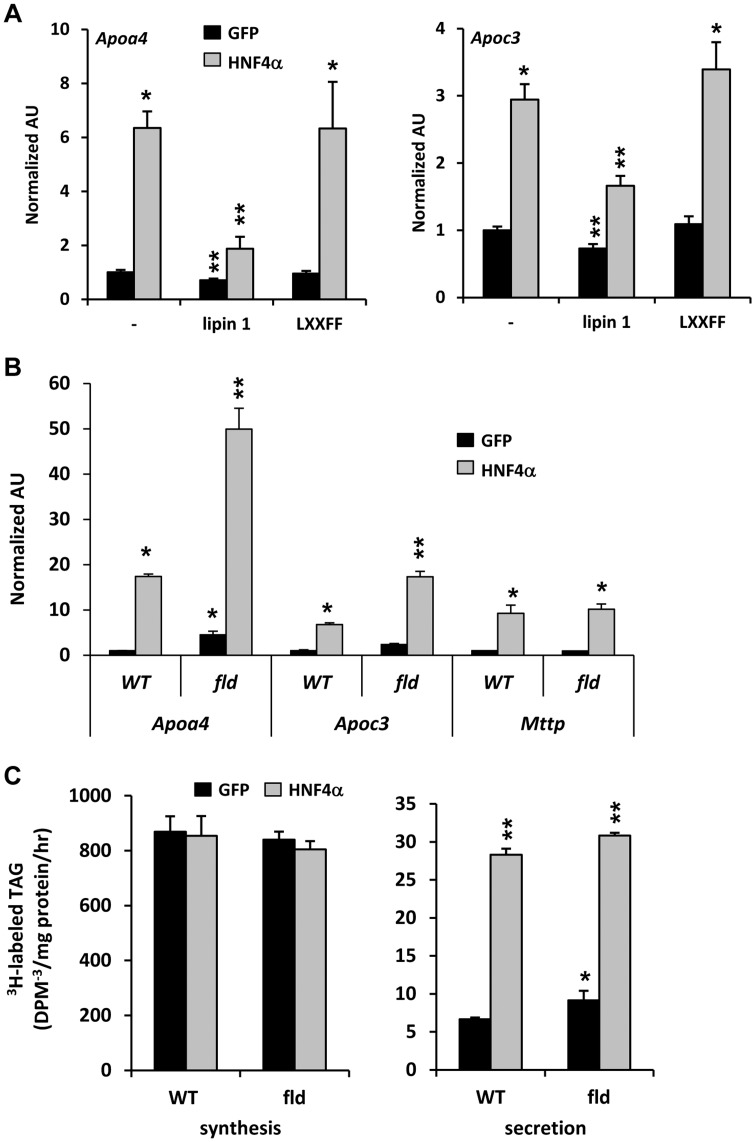
Loss of lipin 1 enhances the effects of HNF4α on apoprotein gene expression. [**A**] Primary hepatocytes were isolated from 6 week old C57BL/6 mice and infected with adenovirus driving expression of GFP or HNF4α in the presence or absence of overexpressed lipin 1β (wild-type or LXXFF). The graphs depict the expression of *Apoa4* and *Apoc3* (n = 5). *p<0.05 versus GFP. **p<0.05 versus GFP control and cells expressing HNF4α alone. [**B and C**] Primary hepatocytes were isolated from 6 week old WT or *fld* mice and infected with adenovirus driving expression of GFP or HNF4α. **[B]** The graph depicts the expression of *Apoa4, Apoc3,* or *Mttp* (n = 5) *p<0.05 versus WT GFP. **p<0.05 versus GFP groups and WT cells expressing HNF4α. **[C]** Graphs depict rates of ^3^H-TAG synthesis and secretion in VLDL. *p<0.05 versus WT GFP. **p<0.05 versus GFP controls.

HNF4α is known to stimulate the expression of various genes involved in VLDL metabolism [29], whereas we have shown that lipin 1 suppresses the expression of these genes [2]. Lipin 1 overexpression suppressed the ability of HNF4α to induce the expression of *Apoa4* and *Apoc3* in an LXXIL motif-dependent manner (Figure 4A). HNF4α overexpression was also more potent at inducing the expression of *Apoa4* and *Apoc3* in *fld* hepatocytes compared to WT controls (Figure 4B). We also assessed rates of TG synthesis and secretion by isolated hepatocytes from WT and *fld* mice and found that, despite the role of lipin 1 in the TG synthesis pathway, rates of TG synthesis were not affected by lipin 1 deficiency or HNF4α overexpression (Figure 4C). Consistent with our previous work [12], rates of VLDL-TG synthesis were significantly increased in hepatocytes from *fld* mice infected with GFP adenovirus (Figure 4C). However, HNF4α-stimulated secretion of newly synthesized VLDL-TG, which was strongly enhanced by HNF4α overexpression, was not affected by loss of lipin 1 (Figure 4C). This may be explained by the strong stimulation of microsomal triglyceride transfer protein (*Mttp*) expression by HNF4α, which is not affected by lipin 1 deficiency (Figure 4B). These data suggest that lipin 1 modulates HNF4α activity to selectively induce fatty acid catabolism whilst suppressing expression of genes encoding apoproteins.

**Figure 5:**
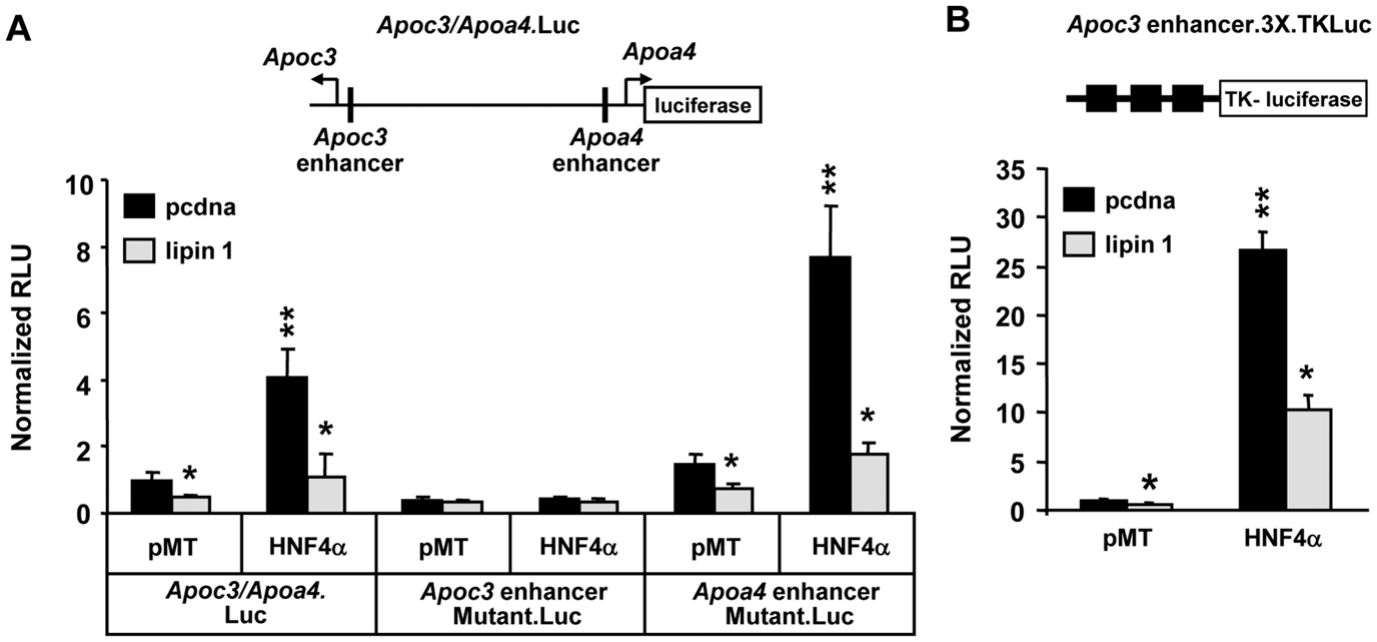
Lipin 1 inhibits *Apoc3/Apoa4* promoter activity in an HNF4α-dependent manner. [**A**] The schematic depicts the luciferase reporter construct under control of the intergenic region between the genes encoding ApoC3 and ApoA4 (*Apoc3/Apoa4.Luc*). The relative positions of two HNF4α response elements denoted as *Apoc3 enhancer* and *Apoa4 enhancer* are indicated. Graphs depict results of luciferase assays using lysates from HepG2 cells transfected with *Apoc3/Apoa4.Luc* reporter constructs and cotransfected with lipin 1 and/or HNF4α expression constructs as indicated. *Apoc3/Apoa4.Luc* constructs were either wild-type or contained mutations in the *ApoC3 enhancer* or *ApoA4 enhancer* HNF4α response elements. The results are the mean of 3 independent experiments done in triplicate. *p<0.05 versus pCDNA control. **p<0.05 versus vector control or lipin 1 cotransfection. [**B**] The schematic depicts the heterologous luciferase reporter construct driven by three copies of the *Apoc3 enhancer* HNF4α response element. Graphs depict results of luciferase assays using lysates from HEK293 cells transfected with *Apoc3 enhancer.3X.TKLuc* and cotransfected with empty vector (pcDNA and pMT), lipin 1, and/or HNF4α expression constructs as indicated. The results are the mean of 3 independent experiments done in triplicate. *p<0.05 versus pCDNA control. **p<0.05 versus vector control or lipin 1 cotransfection.

We sought to explore the molecular mechanism for the crosstalk between lipin 1 and HNF4α using the *Apoc3* and *Apoa4* genes as a model system. These two genes are located adjacent to one another on human chromosome 11 and are oriented in opposing directions so that the promoters and critical regulatory elements that control transcription of both genes are located in a 6 kB intergenic region [30]. HepG2 cells were transfected with a luciferase promoter construct driven by the entire intergenic region between the human *Apoc3* and *Apoa4* genes [17] in the presence or absence of expression constructs for HNF4α and/or lipin 1. As previously reported [16], HNF4α enhanced *Apoc3/Apoa4* promoter activity compared to empty vector control (Figure 5A). Co-transfection of the lipin 1 expression vector significantly repressed basal and HNF4α-induced *Apoc3/Apoa4* promoter activity (Figure 5A).

A site-directed mutation that abrogates binding of HNF4α and other nuclear receptors to a nuclear receptor response element (NRRE) proximal to the *Apoc3* gene (“*Apoc3* enhancer”; [16]) prevented both the lipin 1-mediated suppression and the HNF4α-induced activation of the *Apoc3/Apoa4* promoter (Figure 5A). In contrast, a mutation in another predicted HNF4αRE [16] proximal to the *Apoa4* gene (“*Apoa4* enhancer”) did not influence the effect of either lipin 1 or HNF4α (Figure 5A). The robust HNF4α-mediated activation of a heterologous reporter containing 3 copies of the “*Apoc3* enhancer” was also attenuated by cotransfection of lipin 1β expression vector in HEK-293 cells (Figure 5B).

### Lipin 1 is not Associated with Chromatin in the Apoc3 Promoter

**Figure 6:**
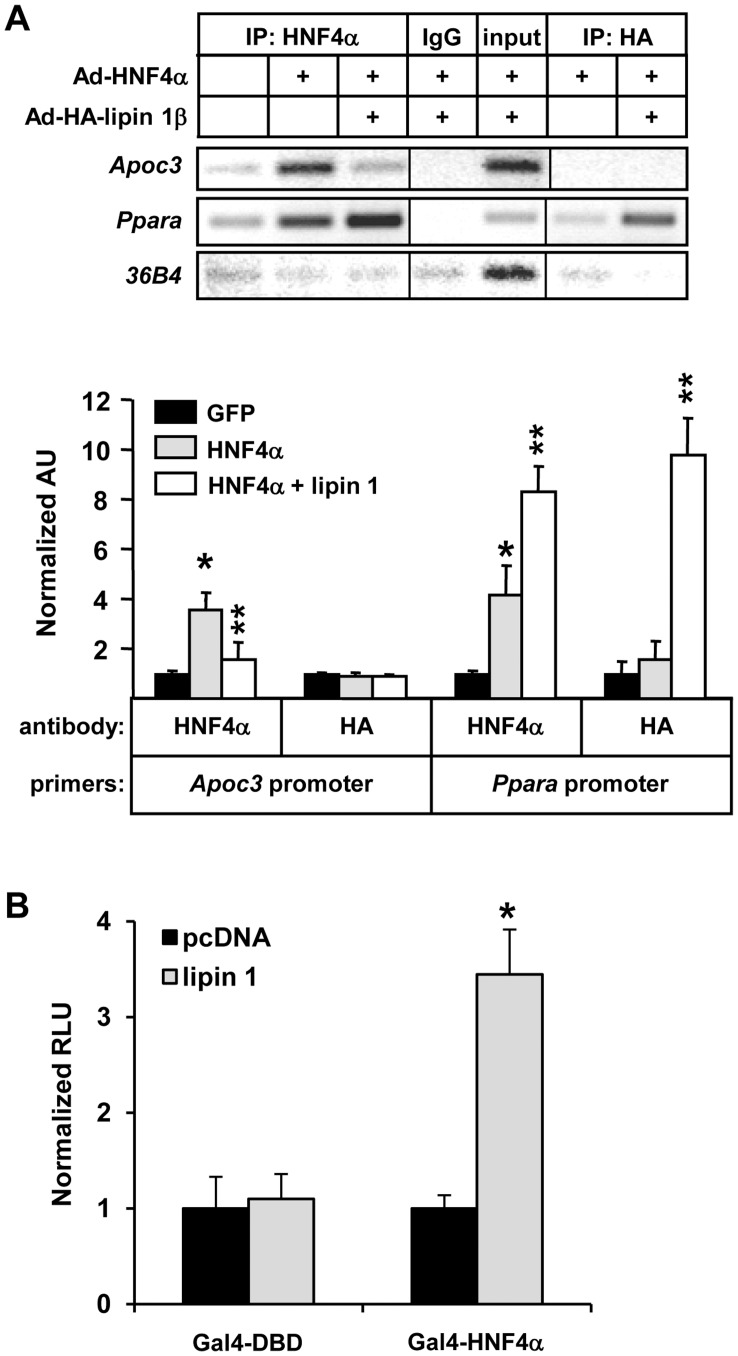
Lipin 1 influences HNF4α promoter occupancy. [**A**] The image depicts the results of ChIP assays using chromatin from HepG2 cells infected with GFP, HNF4α and/or lipin 1β. Chromatin was immunoprecipitated with antibodies directed against HNF4α, the HA tag of lipin 1β or IgG control. Input represents 0.2% of the total chromatin used in the IP reactions. PCR primers were designed to flank the HNF4α response elements in the *Apoc3* or *Ppara* gene promoters. Control primers were designed to amplify the 36B4 gene. The graph depicts results of real-time PCR (SYBR GREEN) to quantify immunoprecipitated chromatin. The results are the mean of 3 independent experiments done in duplicate. *p<0.05 versus pCDNA control. **p<0.05 versus HNF4α alone. [**B**] Graphs depict results of luciferase assays using lysates from HepG2 cells transfected with *UAS*.TKLuc and cotransfected with Gal4-HNF4α or Gal4-DNA binding domain (DBD) control and/or lipin 1expression constructs as indicated. The results are the mean of 3 independent experiments done in triplicate. *p<0.05 versus pCDNA control.

We sought to further dissect the transcriptional regulatory mechanisms mediating the divergent effects of lipin 1 on HNF4α activity. Consistent with the gene expression and promoter assays above, chromatin immunoprecipitation (ChIP) analyses demonstrated that HNF4α occupancy of the *Apoc3* promoter was diminished by lipin 1 overexpression, whereas HNF4α occupancy of the *Ppara* promoter was significantly increased by lipin 1 (Figure 6A). However, ChIP analyses utilizing an antibody to the HA epitope tag of lipin 1 did not detect a significant interaction between lipin 1 and chromatin in the *Apoc3* promoter (Figure 6A). In contrast, significant cross-linking of lipin 1 to the *Ppara* promoter was detected. To examine the effects of lipin 1 on HNF4α intrinsic activity in a promoter-independent fashion, the activity of a Gal4-HNF4α fusion construct on a multimerized Gal4-response element-driven luciferase reporter (UAS-TKLuc) was examined. Lipin 1 overexpression enhanced Gal4-HNF4α activity by more than 3-fold in this mammalian two-hybrid system (Figure 6B). We propose that the suppression of *Apoc3/Apoa4* promoter activity is not mediated via an active repression mechanism and that lipin 1 may influence HNF4α promoter occupancy by directing it towards promoters of genes encoding proteins that affect fatty acid oxidation.

## Discussion

HNF4α is a nuclear receptor transcription factor that is a critical regulator of hepatic gene expression. Previous work has demonstrated important roles for HNF4α in regulating the expression of enzymes involved in VLDL metabolism [16], [31], [32], [33], fatty acid oxidation [18], and a broad profile of genes that define liver development [34]. In this work, we show that the expression of *Lpin1* is also under the control of HNF4α in HepG2 cells and hepatocytes and that this occurs via a direct transcriptional mechanism involving a promoter in the first intron of the *Lpin1* gene. There have been hints in previous studies using ‘omic’ approaches that lipin 1 may be a target gene of HNF4α. *Lpin1* was down-regulated by siRNA against HNF4α and identified in HNF4α ChIP-seq experiments by Bolotin and collegues [35]. In that work, the interaction of HNF4α was generally localized to 3′ to the transcriptional start site of the *Lpin1* gene, which coincides with our findings using promoter luciferase reporter constructs and targeted ChIP approaches. We have also shown that PGC-1α is a critical regulator of lipin 1 expression [10]. HNF4α is also an important partner of PGC-1α for mediating many aspects of the hepatic fasting response; a physiologic condition associated with increased lipin 1 expression [10]. In cardiac myocytes, we have recently shown that PGC-1α coactivates member of the ERR family through these same response elements to induce lipin 1 expression [13]. This suggests that the nuclear receptor partner coactivated by PGC-1α varies depending upon the cell type and expression level of the partners. HNF4α is enriched in hepatocytes, but few other tissues [31]. ERRα and ERRγ expression levels were at or below the edge of detection in HepG2 cells (unpublished observation), but these nuclear receptors are well expressed in muscle cells [27], [36]. Collectively, these data strongly support the idea that lipin 1 is a direct HNF4α target gene in liver cells that is induced in physiologic conditions wherein PGC-1α is activated to coactivate HNF4α.

We have previously shown that lipin 1 and HNF4α physically interact [10], but the physiologic consequences of the interaction and the induction of lipin 1 by HNF4α was not clear. In this work, we showed that increased lipin 1 availability affected HNF4α activity in a pathway-specific manner, suggesting that the activation of lipin 1 serves to feed forward and modulate HNF4α activity. Lipin 1 enhanced HNF4α-mediated activation of fatty acid oxidation while abrogating the ability of HNF4α to induce *Apoa4* and *Apoc3* gene expression. In the nucleus, lipin 1 can function as either a coactivator or corepressor depending upon the context of its transcription factor partner. Lipin 1 is most likely a molecular scaffold that recruits histone acetyltransferases or deacetylases to enhance or repress transcription depending upon the transcription factor partner [10], [11]. However, since ChIP analyses did not detect the presence of lipin 1 on the *Apoc3* enhancer and Gal4-HNF4α activity, a measure of intrinsic activity independent of promoter binding, was enhanced by lipin 1, lipin 1 is probably not inhibiting HNF4α activity by an active repression mechanism. Rather, we surmise that lipin 1 may be mediating this effect by binding to HNF4α and directing its binding to the promoter of one gene versus another. Other coregulatory proteins that act in a promoter-specific manner have been reported. For example, in adipocytes, PGC-1α strongly coactivates PPARγ on the *Ucp1* promoter, but does not enhance expression of *Fabp4* [37], which is also a robust and primary PPARγ target gene [38]. Although it is still unclear how promoter selectivity by coregulatory proteins is mediated, it is possible that the presence of other response elements and DNA-bound transcription factors on certain promoters is required to influence occupancy.

In many ways, the present studies clarify several mechanistic questions from our previous work. For example, although PPARα is coactivated by lipin 1, PPARα deficiency did not affect the transcriptional effects of lipin 1 in liver [10]. The present data indicate that this observation can be explained by the HNF4α-lipin 1 interaction. We also previously reported that *Apoa4* is markedly overexpressed in liver of *fld* mice [39], lipin 1 overexpression suppressed the expression of *Apoc3* and *Apoa4,* and the transcriptional activity of lipin 1 was required for this repressive effect [2]. In this work, we provide a more detailed mechanistic explanation for this observation using the *Apoc3/Apoa4* gene cluster, which is well-characterized as an HNF4α target [16], [31], [40], [41], [42], [43]. However, it is unlikely that the modulation of *Apoc3/Apoa4* expression *per se* is sufficient to explain our previous report that lipin 1inhibits hepatic VLDL-TG secretion [44], [45], [46]. HNF4α overexpression was equally efficacious at increasing VLDL-TG secretion in WT and *fld* hepatocytes, likely because of a strong induction of *Mttp*, which is known to be sufficient to stimulate VLDL secretion. The identification of the gene targets mediating this response will be the subject of future inquiry.

In conclusion, we demonstrate herein that the gene encoding lipin 1 is direct target gene of HNF4α that feeds forward to modulate HNF4α activity, seemingly in a promoter-specific manner. Whereas lipin 1 promotes the expression of genes encoding fatty acid oxidation enzymes in response to HNF4α overexpression, lipin overexpression impedes the induction of apolipoprotein gene expression by this nuclear receptor. These data suggest that lipin 1 functions to promote the catabolic actions of HNF4α, which fits with the induction of lipin 1 in liver of starved mice when rates of fatty acid oxidation are high. These findings also suggest that promoting expression of lipin 1 in liver could help to clear liver fat by promoting its degradation in β-oxidative pathways.
